# Supplementary material for: CtIP forms a tetrameric dumbbell-shaped particle which bridges complex DNA end structures for double-strand break repair
Source: eLife. 2019 Jan 2;8:e42129. doi: 10.7554/eLife.42129 (PMC6344080; doi:10.7554/eLife.42129)
Supplement: Supplementary file 1. — Supplementary Table 1: IC50 values for competitor DNA molecules used in DNA unbinding assays. The reported error is the error associated with the fit to a hyperbolic unbinding curve as shown in Figure 5—figure supplement 2. Supplementary Table 2: Assembly/Source of DNA substrates. Small DNA substrates were prepared by annealing different combinations of short oligonucleotides (A-T). The sequences for the oligonucleotides are presented in Supplementary Tables 3. Supplementary Table 3: Sequences of oligonucleotides used to assemble competitor DNA molecules. [file elife-42129-supp1.docx]

**Supplementary Tables 1-3**

**Supplementary Table 1: IC_50_ values for competitor DNA molecules used in DNA unbinding assays.** The reported error is the error associated with the fit to a hyperbolic unbinding curve as shown in **Figure 5 Figure Supplement 2**.

| **#** | **DNA Substrate*** | **IC_50_** (nM molecules) | **+/- (nM)** |
| --- | --- | --- | --- |
| 1 | Fork  (equivalent to the reference probe DNA) | 55 | 8.7 |
| 2 | Biotin-Fork | 55 | 6.2 |
| 3 | Streptavidin-Biotin-Fork | 28 | 4.4 |
|  |  |  |  |
| 4 | ssDNA (45 bases) | 314 | 56 |
| 5 | Biotin-ssDNA | 360 | 47 |
| 6 | Streptavidin-Biotin-ssDNA | 113 | 13 |
| 7 | Streptavidin ssDNA | 369 | 62 |
|  |  |  |  |
| 8 | dsDNA (45 base pairs) | 392 | 44 |
| 9 | 5'-Biotin-dsDNA | 413 | 58 |
| 10 | Streptavidin-5'-Biotin-dsDNA | 146 | 18 |
| 11 | Double-5'-Biotin-dsDNA | 448 | 105 |
| 12 | Double-Streptavidin-5'-Biotin-dsDNA | 80 | 11 |
|  |  |  |  |
| 13 | 3'-Biotin-dsDNA | 289 | 40 |
| 14 | Streptavidin-3'-Biotin-dsDNA | 142 | 17 |
| 15 | Double-3'-Biotin-dsDNA | 452 | 69 |
| 16 | Double-Streptavidin-3'-Biotin-dsDNA | 78 | 10 |
|  |  |  |  |
| 17 | 5'-3'-Biotin-dsDNA | 306 | 27 |
| 18 | Streptavidin-5'-3'-Biotin-dsDNA | 89 | 23 |
| 19 | Double-5'-3'-Biotin-dsDNA | 410 | 36 |
| 20 | Double-Streptavidin-5'-3'-Biotin-dsDNA | 106 | 15 |
|  |  |  |  |
| 21 | Mid-Biotin dsDNA | 263 | 30 |
| 22 | Streptavidin-Mid-Biotin-dsDNA | 561 | 138 |
| 23 | Double-Mid-Biotin dsDNA | 229 | 33 |
| 24 | Double-Streptavidin-Mid-Biotin-dsDNA | 261 | 42 |
|  |  |  |  |
| 25 | dsDNA (30 base pairs) | 915 | 118 |
| 26 | Streptavidin dsDNA | 861 | 159 |
|  |  |  |  |
| 27 | 3'-Biotin-ssDNA | 384 | 68 |
| 28 | Strepatavidin-3'-Biotin-ssDNA | 263 | 53 |
| 29 | 5'-3'-Biotin-ssDNA | 405 | 44 |
| 30 | Streptavidin-5'-3'-Biotin-ssDNA | 740 | 171 |
| 31 | Mid-Biotin-ssDNA | 780 | 117 |
| 32 | Streptavidin-Mid-Biotin-ssDNA | 678 | 109 |
|  |  |  |  |
| 33 | 5'-overhang | 346 | 47 |
| 34 | 3'-overhang | 305 | 47 |
|  |  |  |  |
| 35 | ssDNA (25 bases) | 2410 | 560 |
| 36 | ssDNA (30 bases) | 2419 | 403 |
| 37 | ssDNA (35 bases) | 831 | 113 |
| 38 | ssDNA (40 bases) | 380 | 34 |
| 39 | ssDNA (45 bases) | 314 | 56 |
| 40 | ssDNA (70 bases) | 86 | 13 |
|  |  |  |  |
| 41 | plasmid dsDNA (pSP73 JY10) supercoiled | 0.54  (4140 nM ntd) | 0.046  360 |
| 42 | plasmid dsDNA (pSP73 JY10) linear | 0.45  (3465 nM ntd) | 0.058  450 |
| 43 | plasmid dsDNA (pSP73 JY10) 45 fragments | 0.63  (4860 nM ntd) | 0.064  495 |
| 44 | φX174 virion ssDNA circular | 0.53  (2880 nM ntd) | 11  495 |
| 45 | φX174 virion ssDNA 15 fragments | 0.51  (2745 nM ntd) | 0.046  360 |

* The constituent oligonucleotides for these DNA substrates are shown in **Supplementary Tables 2 and 3**.

**Supplementary Table 2: Assembly/Source of DNA substrates.** Small DNA substrates were prepared by annealing different combinations of short oligonucleotides (A-T). The sequences for the oligonucleotides are presented in Supplementary Table 3.

| **#** | **DNA Substrate** | **Constituent oligos / source**  **(see also Table 3)** |
| --- | --- | --- |
| 1 | Fork  (equivalent to the reference probe DNA) | A + B |
| 2 | Biotin-Fork | B + C |
| 3 | Streptavidin-Biotin-Fork | B + C |
|  |  |  |
| 4 | ssDNA (45 bases) | D |
| 5 | Biotin-ssDNA | E |
| 6 | Streptavidin-Biotin-ssDNA | E |
| 7 | Streptavidin ssDNA | D |
|  |  |  |
| 8 | dsDNA (45 base pairs) | D + F |
| 9 | 5'-Biotin-dsDNA | E + F |
| 10 | Streptavidin-5'-Biotin-dsDNA | E + F |
| 11 | Double-5'-Biotin-dsDNA | E + G |
| 12 | Double-Streptavidin-5'-Biotin-dsDNA | E + G |
|  |  |  |
| 13 | 3'-Biotin-dsDNA | F + H |
| 14 | Streptavidin-3'-Biotin-dsDNA | F + H |
| 15 | Double-3'-Biotin-dsDNA | H + I |
| 16 | Double-Streptavidin-3'-Biotin-dsDNA | H + I |
|  |  |  |
| 17 | 5'-3'-Biotin-dsDNA | F + J |
| 18 | Streptavidin-5'-3'-Biotin-dsDNA | F + J |
| 19 | Double-5'-3'-Biotin-dsDNA | J + K |
| 20 | Double-Streptavidin-5'-3'-Biotin-dsDNA | J + K |
|  |  |  |
| 21 | Mid-Biotin dsDNA | F + L |
| 22 | Streptavidin-Mid-Biotin-dsDNA | F + L |
| 23 | Double-Mid-Biotin dsDNA | L + M |
| 24 | Double-Streptavidin-Mid-Biotin-dsDNA | L + M |
|  |  |  |
| 25 | dsDNA (25 base pairs) | N + O |
| 26 | Streptavidin dsDNA | N + O |
|  |  |  |
| 27 | 3'-Biotin-ssDNA | H |
| 28 | Strepatavidin-3'-Biotin-ssDNA | H |
| 29 | 5'-3'-Biotin-ssDNA | J |
| 30 | Streptavidin-5'-3'-Biotin-ssDNA | J |
| 31 | Mid-Biotin-ssDNA | L |
| 32 | Streptavidin-Mid-Biotin-ssDNA | L |
|  |  |  |
| 33 | 5'-overhang | F + N |
| 34 | 3'-overhang | D + O |
|  |  |  |
| 35 | ssDNA (25 bases) | P |
| 36 | ssDNA (30 bases) | Q |
| 37 | ssDNA (35 bases) | R |
| 38 | ssDNA (40 bases) | S |
| 39 | ssDNA (45 bases) | D |
| 40 | ssDNA (70 bases) | T |
|  |  |  |
| 41 | plasmid dsDNA (pSP73 JY10) supercoiled | Ref (Yeeles et al., 2011) |
| 42 | plasmid dsDNA (pSP73 JY10) linear | Ref (Yeeles et al., 2011) |
| 43 | plasmid dsDNA (pSP73 JY10) 45 fragments | Ref (Yeeles et al., 2011) |
| 44 | φX174 virion ssDNA circular | New England Biolabs |
| 45 | φX174 virion ssDNA 15 fragments | New England Biolabs |

**Supplementary Table 3: Sequences of oligonucleotides used to assemble competitor DNA molecules**

| **Constituent**  **Oligonucleotide** | **Sequence (5’-3’)** |
| --- | --- |
| A | GCT TGC TAG GAC GGA TCG CTC GAG GTT TTT TTT TTT TTT TTT TTT |
| B | TTT TTT TTT TTT TTT TTT TTC CTC GAG CGA TCC GTC CTA GCA AGC |
| C | Biotin-GCT TGC TAG GAC GGA TCG CTC GAG GTT TTT TTT TTT TTT TTT TTT |
| D | GCT TGC TAG GAC GGA TCG CTC GAG GTT TAC CCT GCT ATA CGG TGA |
| E | Biotin-GCT TGC TAG GAC GGA TCG CTC GAG GTT TAC CCT GCT ATA CGG TGA |
| F | TCA CCG TAT AGC AGG GTA AAC CTC GAG CGA TCC GTC CTA GCA AGC |
| G | Biotin-TCA CCG TAT AGC AGG GTA AAC CTC GAG CGA TCC GTC CTA GCA AGC |
| H | GCT TGC TAG GAC GGA TCG CTC GAG GTT TAC CCT GCT ATA CGG TGA-Biotin |
| I | TCA CCG TAT AGC AGG GTA AAC CTC GAG CGA TCC GTC CTA GCA AGC-Biotin |
| J | Biotin-GCT TGC TAG GAC GGA TCG CTC GAG GTT TAC CCT GCT ATA CGG TGA-Biotin |
| K | Biotin-TCA CCG TAT AGC AGG GTA AAC CTC GAG CGA TCC GTC CTA GCA AGC-Biotin |
| L | GCT TGC TAG GAC GGA TCG C dT-Biotin C GAG GTT TAC CCT GCT ATA CGG TGA |
| M | TCA CCG TAT AGC AGG GTA AAC C dT-Biotin C GAG CGA TCC GTC CTA GCA AGC |
| N | GCT TGC TAG GAC GGA TCG CTC GAG G |
| O | C CTC GAG CGA TCC GTC CTA GCA AGC |
| P | GAG ATA TAC ATA TGA AAA CGC CGT G |
| Q | GCG TAA GCC CCG GGA TGG CTA AAG GCC TTG |
| R | GTT TGT TGT CCT TGA CCA AGT AGA GGC TGC GCT CG |
| S | GGA TTA CCG CTG TGA ATG GGC TTC GTT TTA CGA TTA TTG T |
| T | TTT TTT TTT TTT TTT TTT TTT TTT TTT TTT TTT TTT TTT TTT TTT TTT TTT TTT TTT TTT TTT TTT TTT T |
